# Effect of intimate partner violence on birth outcomes

**DOI:** 10.4314/ahs.v17i3.10

**Published:** 2017-09

**Authors:** Tariku Laelago, Tefera Belachew, Meseret Tamrat

**Affiliations:** 1 Department of Nursing, Hossana College of Health Sciences, Hossana, Ethiopia; 2 Department of Population and Family Health, Jimma University, Jimma, Ethiopia

**Keywords:** Intimate partner violence, birth outcomes, Ethiopia

## Abstract

**Background:**

Violence by intimate partner during pregnancy has many adverse pregnancy outcomes. Thus, that's why we sought to determine association between intimate partner violence during pregnancy and adverse birth outcomes.

**Methods:**

A facility based cross-sectional study was conducted among 183 recently delivered women from March 31–April 30, 2014 in public health facilities of Hossana Town. The data were collected through structured questionnaire and record review. Women who were not mentally and physically capable of being interviewed and those admitted for abortion were excluded. Ethical clearance was obtained from Jimma University. Logistic regression analysis was employed to determine the association between intimate partner violence and adverse birth outcomes.

**Results:**

About 23 % of women experienced intimate partner violence during pregnancy. The result of this study indicated an association of intimate partner violence with low birth weight of the new born (AOR:14.3,95% CI: (5.03, 40.7). Intimate partner violence was not associated with still birth, pre-term birth and Apgar score less than 7 at 5 minutes.

**Conclusion:**

The findings of this study showed that intimate partner violence during pregnancy was associated with a low birth weight of the new born. Health sectors should train health care providers on how to screen, counsel, treat and follow up abused women.

## Background

Intimate partner violence consists the use of physical, sexual or emotional abusive acts and controlling behaviours. An especially concerning form of physical violence during pregnancy is when abusive partners target a woman's abdomen which can cause hurting the woman and potentially jeopardizing the pregnancy^1^,^2^. Intimate partner violence against women is a global public health concern, with 15% to 71% of women experiencing such abuse globally^3^,^4^.

The prevalence of intimate partner violence during pregnancy among ever-pregnant women ranged from approximately 2% in Australia, Denmark, Cambodia and Philippines to 13.5% among ever-pregnant women in Uganda. More than half of the surveys had a prevalence estimate between 3.8% and 8.8%. However; prevalence appeared to be higher in the African and Latin American countries relative to the European and Asian countries though estimates within regions and countries were highly variable^5^. A systemic review of African studies on intimate partner violence against pregnant women reported that the overall prevalence of intimate partner violence during pregnancy ranges from 2.3% to 57.1%. The studies also reported prevalence rates of 23% to 40% for physical, 3% to 27% for sexual and 25% to 49% for emotional intimate partner violence during pregnancy^6^.

Domestic violence is common in Ethiopia in both urban and rural families. About 68% women agree that wife beating is justified in at least one of the specified situations^7^. About 71% of women experienced life time physical and sexual violence by their intimate partner^8^ while 8 % of women experienced intimate partner violence during pregnancy^1^.

Intimate partner violence during pregnancy has been found to be associated with adverse birth outcomes. The risk of being with born low birth weight and pre-term are associated with intimate partner violence. The effect of being born with low birth weight and preterm can result in immediate and long term health and developmental problems. The effects are not limited to women who experience only physical abuse; even psychological abuse has been linked to poor birth outcomes. Physical, mental health problems and negative health behaviours are some of maternal factors that are associated with intimate partner violence during pregnancy^9^,^10^. Studies revealed the long-term effect of intimate partner violence as extremely premature and low birth weight infants. Such children commonly have cognitive deficits, motor delays including cerebral palsy, academic difficulties, language delays, and increased rates of attention problems, behavioural difficulties and psychological problems^11^,^12^,^13^.

A longitudinal study demonstrates that maternal antenatal stress predicts children's behavioural and emotional problems four years later, with the underlying mechanism including the effect of maternal mood on the fetal brain development, which affects the child's behavioural development^14^.

Women who were reporting physical, sexual and emotional violence were more likely to deliver by caesarean section and to have abnormal progress of labour, premature rupture of membranes, low birth weight baby, preterm birth and any hospitalization before delivery than women who did not^15^.

A meta-analysis of 30 studies on maternal exposure to abuse and birth outcomes found that maternal exposure to domestic violence was significantly associated with an increased risk of low birth weight, as well as an increased risk of preterm birth^16^. Contrary to the meta-analysis study, study conducted in Canada identified no statistically significant associations for preterm birth and experience of violence^17^. Similarly, a study conducted in Karachi found the proportion of infants with birth weight < 2500 grams, gestational age < 37 weeks, or 5-minute Apgar < 7 was not significantly greater in abused women than in non-abused women^18^.

Understanding the relationship between violence during pregnancy and adverse birth outcomes could have important clinical and public health implications. Early identification and intervention to prevent violence against pregnant women might reduce adverse birth outcomes. In Ethiopia, information on effects of intimate partner violence on birth outcomes is limited. Therefore, the aim of this research was to determine association between intimate partner violence during pregnancy and adverse birth outcomes among recently delivered women in public health facilities of Hossana Town.

## Methods

### Study area and period

The study was conducted in Hossana Town, which is situated in Hadiya zone of South nation's nationalities regional state of Ethiopia. The Town is located 232 Kilometres far from Addis Ababa. In Hossana Town, there are three governmental health centres, one governmental hospital, and 25 private health facilities. The study was conducted in public health facilities. According to the information of Hadiya zone health department, 2,176 women gave birth in the four public health facilities of Hossana Town in 2013. The proportion of birth attended by health professional in the town was 27% in 2013. The study period lasted from March 31–April 30, 2014.

### Study participants

Women who were giving birth in public health facilities of Hossana Town were recruited as source population for this study. The study population was all women who recently gave birth irrespective of whether the pregnancy outcome was alive or still birth. Women who were not mentally and physically capable of being interviewed and those admitted for abortion or for other pregnancy complications and later discharged undelivered were excluded in this study.

### Data collection and measurement

Facility based cross sectional study was conducted among 195 recently delivered women in public health facilities of Hossana Town. The required number of sample was allocated proportionally among the four public health facilities. Consecutive sampling technique was undertaken by taking every woman who was presented in selected health facilities until allocated sample size was reached.

Pre-tested structured questionnaire and record review checklist were used to collect data from the study subjects. The questions were adopted from WHO multi-country study on domestic violence^8^. Record review checklist was developed by principal investigator to collect data on new born birth outcomes. The questionnaire was first prepared in English, translated into Amharic, and then re-translated back to English to check for its consistency. Pre-test was carried out among ten study subjects who were not included in the actual study. Based on the result of pre-test, appropriate modifications were made to have the final version.

The data collection instrument comprises of socio-demographic and economic factors, experience of intimate partner violence during pregnancy, and record review checklist. The record reviews were conducted on delivery summary book of each respondent. The data collected on new born outcomes included alive birth, still birth, birth weight, Apgar score at 5 minutes, term and preterm birth.

Four female nurses and two supervisors' were recruited as interviewers and as supervisors respectively. Data collectors and supervisions were trained for one day on interviewing techniques, purpose of the study and ethical aspects.

The principal investigator and supervisor made a day to day on site supervision during the whole period of data collection and checked each questionnaire daily for its completeness and consistency.

Intimate partner was considered as current husband, co-habited (live in the same house without formal marriage), or boyfriend. Prevalence of intimate partner violence during pregnancy was measured by any act of physical, sexual or psychological abuse during last pregnancy by intimate partner.

We defined intimate partner violence according to the WHO Multi-Country Study^8^, as physical violence includes any of one or more (slapped, pushed or shoved, hit with fist or something else that could hurt her, beaten abdomen, choked or burnt on purpose, used or threatened to use knife, gun or weapon).

Sexual violence includes any of one or more (forced into sexual intercourse when she did not want, had sexual intercourse when she did not want to because she was afraid of what partner might do, forced to do something sexual that she found degrading or humiliating).

Psychological violence includes any of one or more (insult, humiliation, intimidated on purpose, threatened to hurt her or someone she cared about). Overall prevalence of intimate partner violence was measured by any act of physical, sexual or psychological violence during last pregnancy.

Adverse birth outcomes were measured by low birth weight, preterm birth, still birth or Apgar score <7 at 5 minutes. Low birth weight was defined as a live birth weighing < 2500 grams. Preterm was defined as a neonate born before 37 completed weeks. The cut off points used for the birth outcomes were based on standards^19^. Planned pregnancy was assessed by asking women whether her last pregnancy was planned by her and her partner. Recently delivered women were defined as women who were within 7 days of post-delivery in public health facility of Hossana Town.

### Data processing and analysis

Data were entered into Epi-data version 3.1 20, then, it was exported into SPSS version 16.0 statistical software for further analysis^21^. Frequency tables and descriptive summaries were used to describe the study variables. Bivariate logistic regression analysis was used to see significance of association between the outcome and independent variables. Variables with P-value < 0.05 on bivariate analysis were transferred to multivariate logistic regression. Odds ratios at 95% CI were computed to measure the strength of the association between the outcome and the independent variables.

Multivariate logistic regression was performed to assess the relationship between intimate partner violence during pregnancy and adverse birth outcomes with adjustments for potential confounders. Confounding was assessed by entering potential confounders into a logistic model at a time, and then compared the unadjusted and adjusted ORs. We considered the following variables as possible confounders in these analyses. These were age of women, planned pregnancy, educational status, occupation, average monthly income, number of children, number of pregnancies and marital status. Final logistic regression models included covariates that altered unadjusted ORs by at least 10%^22^. P-value < 0.05 was considered as a statistically significant

### Ethical consideration

We followed World Health Organization ethical and safety recommendations^23^. Ethical clearance was taken from ethical clearance committee of the Jimma University. Informed oral consent was taken from the respondents. The oral (non-written) consent was obtained because written consent demanded a certain level of education to read and sign the consent. The consent was documented by asking if the respondents want to participate in the study or not. The respondents were assured that they have full right to participate or withdraw from the study. The consent procedure was assured by ethical committee of Jimma University.

Interviewers were trained to be aware of the effects that the questions may have on informants and how best to respond, based on a woman's level of distress. Privacy and confidentiality of the respondents were protected.

## Results

Of the 195 recently delivered women, 183 (94 %) gave an informed consent and were included in the study. The mean age women were 25.4 (SD ±4.6). Regarding to educational status, 64(35%) attained primary education. Occupation status of respondent's showed that house wife accounted 98(53.6%). One hundred seventy four (95%) of women were married. Ninety two (50.3%) of respondent became pregnant three or more times. For majority of women, 137(75%) their last pregnancy was planned. More than half of respondents 114(62.3%) had one to three children. The mean children women had were 2.8(SD±1.9). The median monthly income of the respondent's was 1200 Ethiopian birr (table 1).

**Table 1:** Characteristics of the study population, Hossana, April 2014.

	Intimate partner Violence(n=43)		No intimate partner violence(n=140)	
Variables	Frequency	Percentage	Frequency	Percentage
**Age groups(n=183)**				
18–24	14	32.6	61	43.6
25–29	14	32.6	53	37.9
30–34	10	23.3	18	12.8
35–38	5	11.6	8	5.7
**Educational status(n=183)**				
No formal education	12	27.9	21	15
Primary education(grade 1–8)	17	39.5	51	36.4
Secondary education(grade 9–12)	8	18.6	34	24.3
Tertiary education(Above 12 grade)	6	14	34	24.3
**Occupational status(n=183)**				
Housewife	27	62.8	71	50.6
Student	3	7	14	10
Merchant	5	11.6	25	17.9
Government employee	7	16.3	26	18.6
Non-government employee	0	0	3	2.1
Other*	1	2.3	1	0.7
**Marital status(n=183)**				
Married	40	93	134	95.7
Never married	1	2.3	4	2.9
Divorced	2	4.7	2	1.4
**Number of pregnancy(n=183)**				
One or two	15	34.9	76	54.3
Three or above	28	65.1	64	45.7
**Last pregnancy** **planned(n=183)**				
Yes	19	44.2	118	84.3
No	24	55.8	22	15.7
**Number of children(n=180)**				
No child	0	0	1	0.7
One to three child	20	47.6	100	71.9
Four and above	22	52.4	38	27.3
**Average monthly** **income(n=180)**				
<1200 Ethiopian birr	29	67.4	70	51.1
≥1200 Ethiopian Birr	14	32.6	67	48.9

* daily workers

Forty three (23%) of women experienced at least one form of intimate partner violence during pregnancy. Psychological violence was the most common form 36 (20%) followed by physical 27(15%) and sexual 22 (12%). From physical violence, slapping 27(14.8 %) was the commonest form of violence. Physically forced to have sexual intercourse 22(12%), and insulting 36(19.7) were commonest form of sexual and emotional violence respectively (Table 2).

**Table 2:** Forms of intimate partner violence during pregnancy

Forms of violence (n=183)	No	Percent
All forms	43	23
Physical	27	15
Sexual	22	12
Psychological	36	20

## Birth outcomes

One hundred eighty three records were reviewed to obtain the birth outcomes of the newborn. Of the 183 births, 175(95.6%) were live births and 8(4.4%) were still births. Of the live births, 39 (22.3%) were low birth weight. Only 16(8.7%) of the new born were preterm. Nine (5%) of new born Apgar scores were less than 7 at 5 minutes. From the total 183 births, 47(25.6%) had at least one adverse birth outcomes and sixteen (34%) had more than one adverse birth outcomes (Table 2).

Association between intimate partner violence during pregnancy and adverse birth outcomes.

Bivariate association was done using enter method to identify association between intimate partner violence during pregnancy and adverse birth outcomes. On bivariate analysis, intimate partner violence during pregnancy showed significant association with low birth weight (COR=16.3(6.6, 40). There was no association between IPV and still birth (COR=0.29(0.07, 1.12), Apgar score less than 7 at 5 minutes (COR=1(0.2, 5.0) and preterm birth (COR=0.36(0.12.1.014).

A multivariate analysis was done to see significant association between intimate partner violence during pregnancy and low birth weight. After adjustment for age of women, number of pregnancy, planned pregnancy, educational status of women and monthly income, intimate partner violence during pregnancy was associated with low birth weight. The new born whose mothers were abused during pregnancy were 14.3 times more likely to be having low birth weight as compared to new born whose mothers were not abused during pregnancy (AOR=14.3(5.1,40.7) (Table 3).

**Table 3:** Association between birth outcomes and intimate partner violence during pregnancy

Birth outcomes	Violence (n=43)	No violence (n=140)	COR (95% CI)	AOR (95% CI)**
**Low birth weight**				
Yes	22(56.4)	17(43.6%)	16.3(6.6,40)*	14.3(5.1,40.7)*
No	10(7.3%)	126(92.7%)	1	1
**Preterm birth**				
Yes	7(43.8)%	9(56.2 %)	0.36(0.12,1.014)	-
No	36(21.6 %)	131(78.4 %)	1	
**Apgar score less than 7** **at 5 minutes**				
Yes	2(22.2%)	7(77.8)	1(0.2,5.0)	-
No	37(22.3%)	129(77.7%)	1	
**Still birth**				
Yes	4(50%)	4(50%)	0.29(0.07,1.12)	-
No	39(22.3%)	136(77.7%)	1	

* p-value<0.05

** Adjusted for the following variables: age of women, number of pregnancy, pregnancy planned or unplanned, educational status of women and monthly income.

## Discussion

The overall prevalence of intimate partner violence during pregnancy in current study was 23%. The psychological violence was the most common form (20%) followed by physical (15%) and sexual (12%). This finding is consistent with studies done in other African countries. A review of literature on IPV in African countries found that its prevalence ranges from 2% to 57 %. The overall prevalence of intimate violence during pregnancy in current study is lower than Kenyan study, which showed 37% of women were abused during pregnancy^6^,^24^. The difference may be due to the difference in culture and norms between the countries.

The prevalence of intimate partner violence during pregnancy in current study is higher than the WHO study of Ethiopia, which revealed 8% of women were abused during pregnancy^1^. The difference may be due to difference in study setting and populations' characteristics.

The current study has publicized that there is significant association between intimate partner violence during pregnancy and low birth weight of newborn. Studies done in United States of America on maternal exposure to abuse and birth outcomes showed that there was an association of intimate partner violence and low birth weight^9^,^16^,^25^. Similarly, studies done in Iran found an association between violence and low birth weight^18^. Other studies have also exhibited consistent result as current study^26^,^27^.

The pathway through which violence may lead to risk of adverse birth outcomes has not been fully understood. One possible pathway is the direct effect of blunt physical trauma and subsequent adverse pregnancy outcome^28^. A second potential pathway is elevated maternal stress levels and poor nutrition. Thus, these two factors are associated with low birth weight or preterm delivery and are well known risk factors for perinatal and infant mortality^29^–^31^.

On the other hand, our study is inconsistent with the study done in Pakistan^18^ and Unites States of America, which presented that domestic violence during pregnancy, was not associated with low birth weight^32^. The differences may be due difference in study design and difference in population characteristics.

In current study, Apgar score less than 7 at 5 minutes, still birth and preterm birth were not greater in abused women than non-abused women. This finding is in line with study in Pakistan, which showed that proportion of infant with Apgar score less than 7 at 5 minutes, still birth and preterm birth was not significantly greater in abused women than non-abused women^18^. Similarly, Canadian and USA study found no statistically significant associations of preterm birth and violence^17^,^32^,^33^.

Contrary to current study, study done in Peru and meta-analysis of 30 studies found maternal exposure to domestic violence was significantly associated with an increased risk of preterm birth^10^,^16^,^34^. Previous study also showed the increased odds of low Apgar scores in infants of abused women^35^. A research finding suggests that perinatal death (i.e., deaths in the first week of life and fetal deaths/stillbirths) is more common among women who experience violence during pregnancy^34^. The discrepancies may be related to the large sample size of previous study. It may also be related to the difference in study setting, study population, and difference in operational definition of intimate partner violence.

## Limitation of the study

Since this study was a cross sectional in design, it is difficult to establish causes and effect relationships among outcome of interest and explanatory variable. The sensitive nature of the topic and its proneness to response bias may lead to under-reporting of the true extent of the abuse.

Since we collected data from a secondary source there may be quality problems. Factors that can influence birth outcomes such as antenatal follow up, alcoholism, smoking and substance abuse were not collected in the survey, therefore adjusting for these factors was not possible.

Since the study was facility based and women who delivered at home were excluded in this study. Because of this reason, applicability of findings to the general population is uncertain. Besides, the wide confidence intervals were observed in this study and it may affect precision. This may be due to small sample size. Hence, interpretation of the finding shall take in to account all the factors mentioned above.

## Conclusion

The findings of this study revealed that intimate partner violence during pregnancy is associated with adverse birth outcome (low birth weight of new born). Thus, health sectors should train health care providers on how to screen, counsel, treat, and follow up of abused women. The study further calls for longitudinal research regarding the pathway through which violence may lead to risk of adverse birth outcomes.
